# Ben Katz Will “Mass Spec Everything” to Figure out What Is in Our Food

**DOI:** 10.1021/acscentsci.6c00954

**Published:** 2026-06-16

**Authors:** Victoria Atkinson

## Abstract

His work has revealed a lack of transparency in fruit- and vegetable-based products.

## Graphical abstract

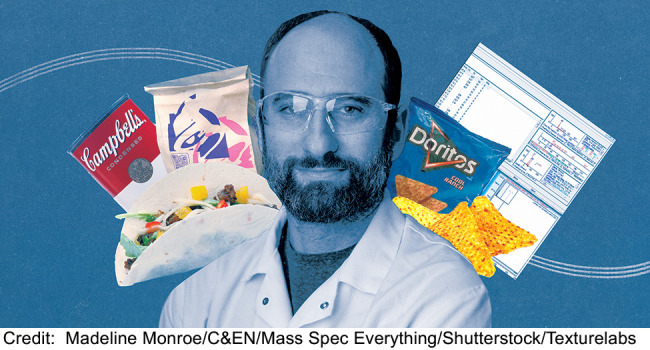

“I wonder if there’s any real meat in a Taco Bell?” mused Ben Katz while strolling around the University of California, Irvine, campus during a sunny lunch break back in 2023. No doubt many of us have pondered similar questions while chowing down on fast food, but as a development engineer at the mass spec facility at UC Irvine, Katz was in the unique position of being able to find out for himself.

**Figure:**
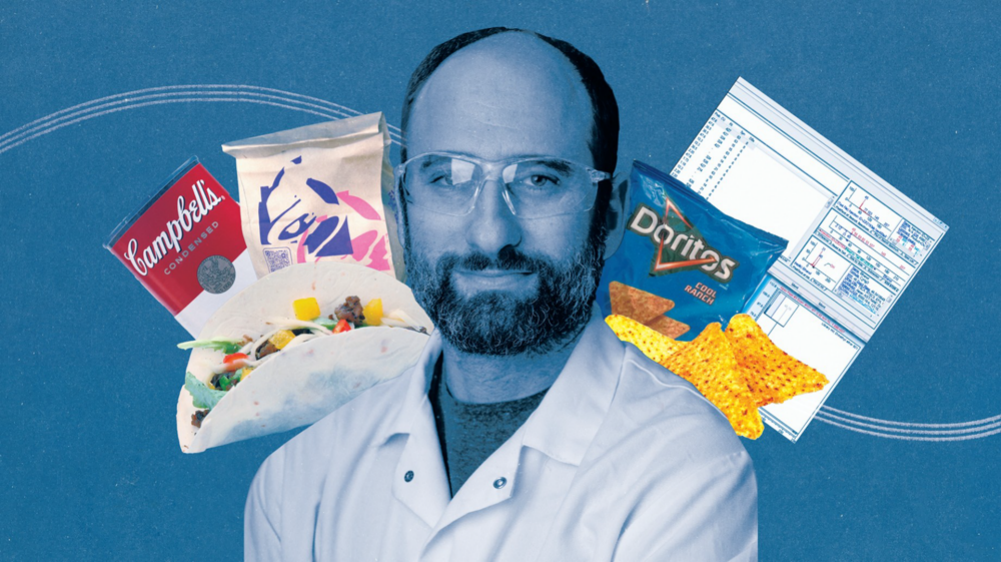
Credit: Madeline Monroe/C&EN/MassSpecEverything/Shutterstock/Texturelabs. Representative processed food images shown for illustrative purposes.

Using the high-performance equipment in his analytical suite, he ran a quick round of experiments and identified both carnosine and carnitine—two tell-tale molecules proving that his lunch was indeed meat. He shared the whimsical investigation in a short TikTok video and thought nothing more of it.

When he looked at the video the next day, it had racked up tens of thousands of views, eventually breaking 100,000. Thus, the comedy-educational social media brand MassSpecEverything was born. Katz now regularly posts videos exploring the hidden ingredients in all manner of everyday products, including a recent collaboration with YouTuber LabCoatz to reverse engineer the top-secret recipe for Coca-Cola.

But while these bizarre samples make for great entertainment, a much more serious and scientific reason exists behind this unconventional analysis, Katz says.

The mass spec facility is shared across multiple departments at UC Irvine, and a huge part of Katz’s job is developing new methods to analyze challenging samples, be it a new skin-cream formulation given to him by medical engineers or a Renaissance-era paint flake from art historians. “The student’s sample is usually so precious that I can’t do method development on it,” Katz explains. “Instead, I’ll try to find a similar molecule class or matrix, testing new methods on foods or cosmetics. It’s actually allowed me to get a lot better at analyzing different molecule classes.”

After the early success of his Taco Bell video, he realized these relatable everyday samples were attracting a new audience eager to understand what’s in their food and why. He began to include a detailed commentary alongside the analysis, theorizing how the production process might change the food’s chemical profile. Surprisingly, fruit and vegetable products turned up the most remarkable results.

Having worked within the agricultural industry prior to joining UC Irvine, Katz is intimately acquainted with the delicate balance between food security, sustainable farming practices, and production economics. He was astonished by the widespread lack of transparency he uncovered while comparing different brands of hot sauce.

He had set out to see which sauce contained the most capsaicin and whether any contained synthetic colors, but “I kept coming across these weird synthetic compounds—polyethoxylated tallow amines and diphenylamine [DPA],” Katz says. “I went on a deep dive, and these are used as coatings on the outer surface of produce. They’re designed to never fall apart, so they follow through the whole manufacturing process, into products like hot sauce.”

**Figure:**
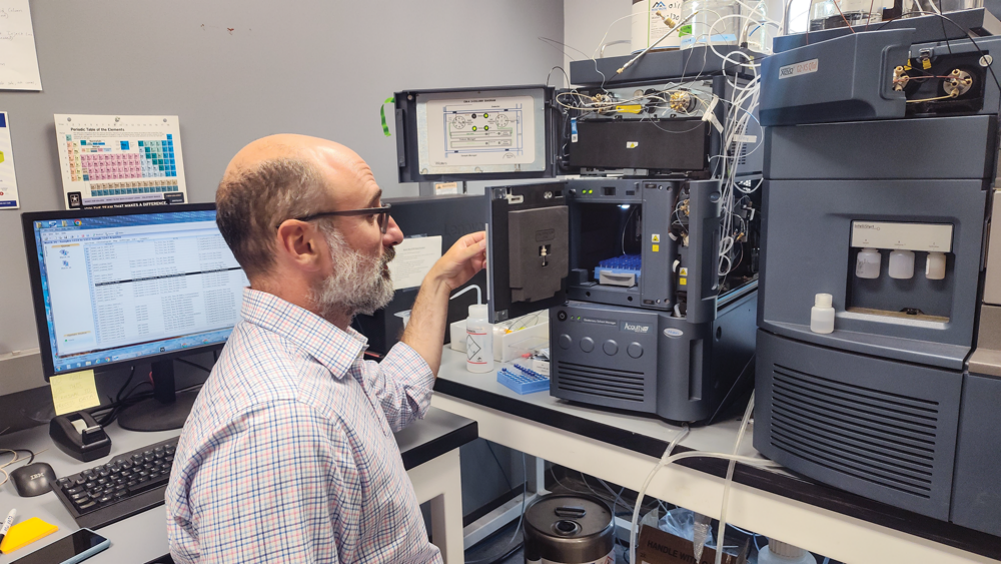
Ben Katz loads samples into a quadrupole time-of-flight mass spectrometer. Credit: Ben Katz.

Because they are added during the initial farming and harvesting stage, both chemicals are considered carryover additives, and US food producers are therefore not required to include them in ingredient listings. But digging a little deeper, Katz discovered a raft of ethical issues associated with their use, raising questions of why consumers haven’t been told they could be ingesting them.

“DPA is a fruit scald inhibitor. It stops apples, etc. getting brown spots after they’re taken from cold storage,” he explains. In the US, this compound is sprayed onto almost all apples and pears at the point of harvest. It persists in the food production supply chain and eventually ends up in finished products such as applesauce and fruit juice. This practice has attracted heavy criticism from US advocacy groups, particularly the Environmental Working Group, which publishes an annual “Dirty Dozen” list highlighting the agricultural products that contain the highest levels of pesticide and chemical residues.

Both the European Union and UK took more-decisive action and banned DPA in food production more than 10 years ago, owing to concerns around toxicity and potential breakdown into carcinogenic nitrosamines. “It’s an entirely cosmetic additive, so there isn’t any reason not to inform people,” Katz says. “It should be advertised, and consumers should have the option to make the correct decision for themselves.”

The implications of these hidden chemicals are even more complex for polyethoxylated tallow amines, greasy surfactants that are made from animal fats. The compounds are used as a solvent in glyphosate pesticide formulations and ensure that the polar active ingredient remains coated on a crop’s leaves. But because tallow is derived from animals, glyphosate-treated fruits and vegetables aren’t technically vegetarian, creating an ethical quandary for anyone with moral or religious dietary requirements.

Consumers should be made aware of animal-based additives, Katz says, because access to this information would enable individuals to make choices that better suit their lifestyles. Katz thought at the time that consumers could buy organic products to avoid synthetic pesticides and thus tallow amines. But his insider insight left him with the niggling feeling that he hadn’t uncovered the whole story.

“It got me wondering, Is there any situation where you can have an organic label on something, but it still has polyethoxylated tallow amines on it?” Katz says. “And the answer is, yes, all the time!”

As Katz explains in a 2024 video, producers can file a petition with the US Food and Drug Administration for an exemption from the usual organic regulations if a crop emergency occurs, such as a disease outbreak or a pest infestation. In these emergency cases, organic farms may use chemical pesticides on specified crops without losing their organic certification.

The farmers can’t label the sprayed crops as organic. But the pesticide compounds are mobile and can potentially migrate to other crops on the farm, crops that *will* be labeled organic, Katz says. By searching government records with the help of an artificial intelligence chatbot, he uncovered exemptions on almost every type of US crop over the previous 4 years.

“It’s a difficult one. This isn’t about demonizing the food industry. If they’re doing this many organic exemptions every year, it seems like it’s an essential excipient,” he says. “At the same time, I’m bringing attention to this because it’s ridiculous that we’re giving all these certifications, and then they may not even mean anything.”

Ultimately, the issue comes down to transparency. People have a right to know what is in their food, and Katz hopes that shining light on additives through his comedy channel will encourage more people to push back and pressure industry to develop cleaner alternatives.

*Victoria Atkinson is a freelance contributor to* Chemical & Engineering News, *the independent news outlet of the American Chemical Society.*

